# Stress enhances model-free reinforcement learning only after negative outcome

**DOI:** 10.1371/journal.pone.0180588

**Published:** 2017-07-19

**Authors:** Heyeon Park, Daeyeol Lee, Jeanyung Chey

**Affiliations:** 1 Department of Psychology, Seoul National University, Seoul, Korea; 2 Department of Neuroscience, Department of Psychiatry, Yale School of Medicine, New Haven, United States of America; 3 Department of Psychology, Yale University, New Haven, United States of America; Centre national de la recherche scientifique, FRANCE

## Abstract

Previous studies found that stress shifts behavioral control by promoting habits while decreasing goal-directed behaviors during reward-based decision-making. It is, however, unclear how stress disrupts the relative contribution of the two systems controlling reward-seeking behavior, i.e. model-free (or habit) and model-based (or goal-directed). Here, we investigated whether stress biases the contribution of model-free and model-based reinforcement learning processes differently depending on the valence of outcome, and whether stress alters the learning rate, i.e., how quickly information from the new environment is incorporated into choices. Participants were randomly assigned to either a stress or a control condition, and performed a two-stage Markov decision-making task in which the reward probabilities underwent periodic reversals without notice. We found that stress increased the contribution of model-free reinforcement learning only after negative outcome. Furthermore, stress decreased the learning rate. The results suggest that stress diminishes one’s ability to make adaptive choices in multiple aspects of reinforcement learning. This finding has implications for understanding how stress facilitates maladaptive habits, such as addictive behavior, and other dysfunctional behaviors associated with stress in clinical and educational contexts.

## Introduction

Reward-seeking behaviors can be described by two different computational principles that might be supported by distinct neuroanatomical substrates [1–4]. On the one hand, a goal-directed controller selects behaviors expected to produce the best outcomes according to the knowledge of the decision-maker’s environment and motivational state. The process by which the knowledge is updated and outcomes expected from alternative actions are derived from this knowledge is referred to as model-based reinforcement learning (RL). On the other hand, a habit controller relies on the expected values of outcome adjusted incrementally by trial and error, and results in automatic and less computationally demanding action selection. Accordingly, these goal-directed and habit systems might favor different actions, when the motivational status of the actor or the properties of environment change rapidly. However, precisely how the balance between these two controllers is adjusted across different behavioral settings remains poorly understood.

Stress might influence the arbitration between a goal-directed and a habit controller during decision making. Previous studies showed that stress causes humans to repeat behavior previously learned despite environmental changes [5–9] and tends to impair episodic memory while enhancing sensory processing [10–12], raising the possibility that stress might promote a switch from a high-order cognitive control to a simpler stimulus-response mapping. However, these previous studies have not examined precisely whether and how different aspects of learning and action selection are influenced by stress in a dynamic environment.

One of the critical issues regarding the relationship between stress and decision-making is how stress has an impact on the trade-off between habit and goal-directed behaviors. More specifically, whether stress leads to more habitual behaviors by either selectively weakening the process of goal-directed behaviors, by merely strengthening the process of habit or both. It is also possible that the effect of stress on behavior might vary depending on whether the result of previous behavior was positive or negative. Indeed, previous studies have suggested that stress might differently influence decisions depending on the valence of the outcome [13–15]. Considering that the neural circuits of reward processing frequently reflects valence-dependent activity of outcome [16, 17], it is possible that stress alters the neural processing of reinforcement and punishment differentially. In other words, stress may boost the neural signals related to decision-making differentially depending on the valence of outcomes. Finally, it remains unclear whether persistent behaviors resulting from stress simply reflects a decrease in the ability to incorporate the information about environmental changes, as quantified by the rate of learning, rather than changes in the nature of RL itself.

In the present study, we investigated the effects of stress on multiple aspects of RL such as model-free and model-based tendency according to the valence of the outcome and the learning rate. Participants were assigned to either a stress or a control condition before performing a multiple-stage decision-making task designed to distinguish model-based behavior from model-free RL behavior. In this task, reward probabilities associated with different choices were periodically reversed. By applying computational models to choice data, we quantified the extent to which choices were influenced by model-free vs. model-based RL, and dissociated the RL processing according to whether decision was followed by positive or negative outcome. Also, how the learning rate was affected by stress was examined.

## Materials and methods

This study was approved by the Seoul National University Institutional Review Board (SNUIRB), and all participants provided written informed consent.

### Participants

Fifty six healthy undergraduate students participated in this study (29 women, 27 men; age, 20.36 ± 1.91; body mass index 21.00 ± 2.52). Individuals who met any of the following criteria were excluded from participation: history of head injury, treatment with psychotropic medications, steroids, or any other medication that affects the central nervous system or the endocrine systems, current medical illness, self-report of mental disorder or substance abuse, existence of current stressful episode or major life event. Also, smokers and women taking oral contraceptives were excluded from the study due to possible effects of nicotine and oral contraceptive on the neuroendocrine stress response [18, 19]. Although gender could affect the hypothalamus-pituitary-adrenal cortex responsiveness to psychosocial stress differently, it has been demonstrated that there were no differences in salivary cortisol response between men and women in the luteal phase [18]. Therefore, women in the late luteal phase (after Day 21 and before the start of the next cycle) of the menstrual cycle were included in this study. Participants were asked to refrain from caffeine and physical exercise during the 6 hours prior to participation, and then were randomly assigned to the stress and the control conditions. Age (*t*_50_ = 1.23, *p* = .226), body mass index (*t*_50_ = -.20, *p* = .846), and perceived stress during the past month (*t*_50_ = .71, *p* = .483), assessed with Perceived Stress Scale [20], were not significantly different between participants in the two conditions. Four participants (two from each condition), who continued to choose the same action in more than 95% of the trials during the task, were excluded from the analysis, since this reflected lack of learning.

### Stress protocol

The socially evaluated cold pressor test (SECPT) [5, 21] was administered to the participants in the stress condition (15 women and 13 men). They immersed one hand (left-handed, right; right-handed, left) up to and including the wrist for 3 minutes (2 participants did it for 2 minutes which was their limits) into ice water (0 ~ 2^°^C). During hand immersion, they were recorded on video by an unfamiliar person. Participants in the control condition (14 women and 14 men) submerged one hand up to and including the wrist for 1 minute in warm water (36 ~ 38^°^C), and they were not recorded on video. To assess whether the treatments were successful, participants were required to report subjective stress on the visual analogue scale (VAS), with the lower and upper bound of the scale marked with numbers 0 and 100, representing a range from “no stress” to “the most stressful.” All experiments took place between 1:00 P.M. and 5:40 P.M. to control for diurnal rhythm of the stress hormone (cortisol). Ten minutes after the cessation of the SECPT or the control procedure, the participants performed a two-stage reversal learning task described below.

### Behavioral task

**Fig 1:**
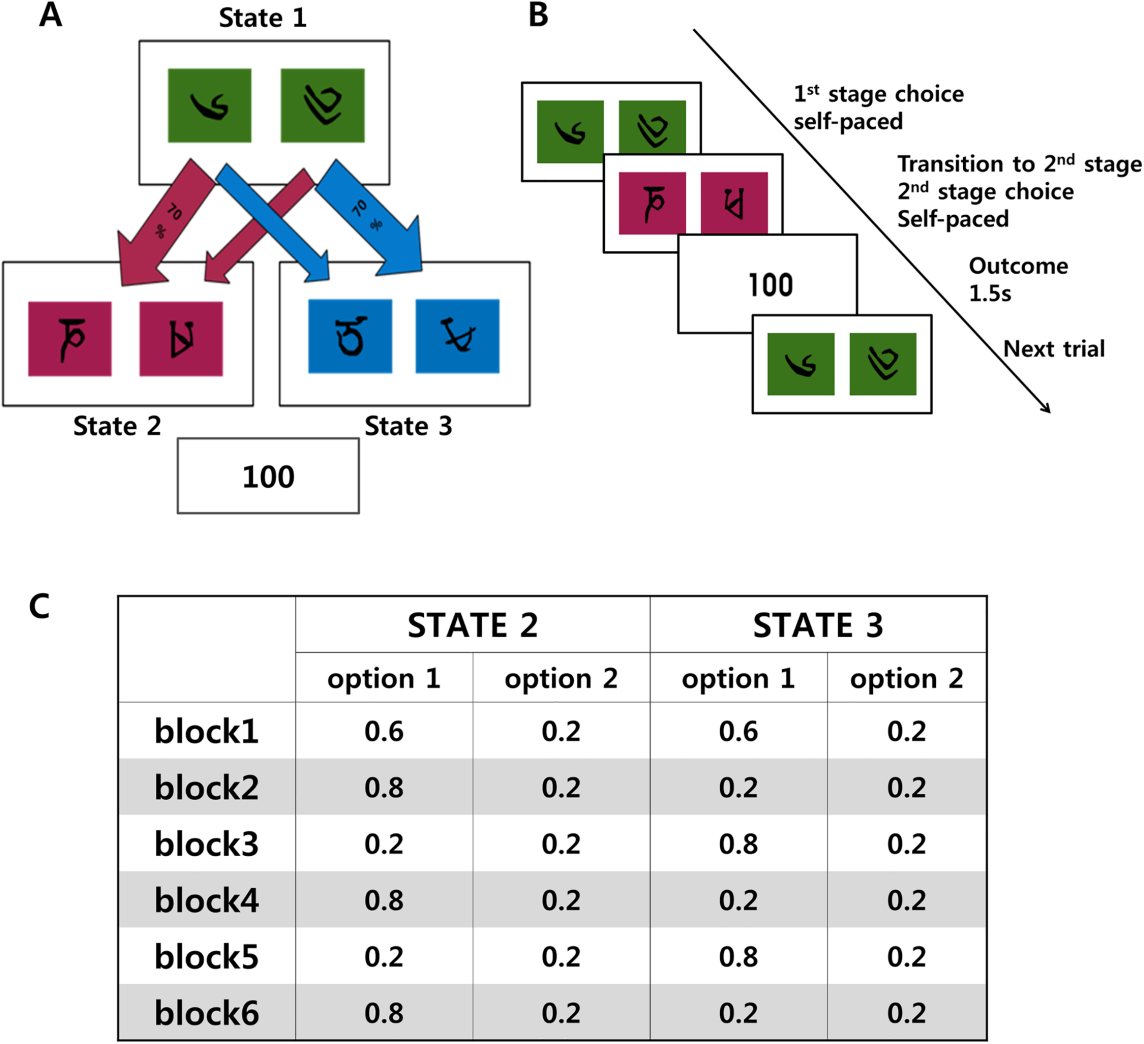
Task design. (A) Task structure. Choice in the first stage leads probabilistically to different states in the second stage. Each stimulus in the second-stage resulted in either 0 or 100 points with different probabilities. (B) Timeline of events in a single trial. (C) Reward-probabilities of four options in stage 2.

We used a two-stage reversal learning task which combined a reversal learning paradigm with the two-stage Markov decision task developed by Daw and his colleagues [22] (see Fig 1 for details). The two-stage Markov decision task has been used to distinguish the contribution of model-free and model-based RL to action selection. We also adopted the reversal learning paradigm, so that the participants were faced with a changing environment, and their choices in response to discrete environmental change could be investigated. The task consisted of six blocks of 40 trials, totaling 240 trials without any breaks. There was no explicit cue for block transition.

Each trial required two successive choices. In the first stage (“state 1”), participants chose between two options, represented by figures similar to Tibetan characters in green-colored boxes. The first-stage choice led probabilistically to one of the two second-stage states (“state 2” and “state 3”), represented by different colors (pink and blue). Each of the first-stage options was associated strongly (with a chance of 70%) with one of the two states in the second-stage, and this contingency was fixed throughout the experiment (Fig 1A). In the second-stage, subjects made another binary choice, and this second choice was linked to either 100 or 0 points depending on reward probability that was predetermined (Fig 1C). The assignment of two colors (pink or blue) to state 2 and 3 was counterbalanced across subjects, and the locations of two options in each state were randomized from trial to trial.

The reward probabilities for the two options in the second stage changed from block to block, employing the reversal learning paradigm as shown in Fig 1C. In the first block, both states 2 and 3 had one option leading to 60% chance of reward while the other leading to 20% chance. Therefore, in block 1, the two options in the first stage were equally favorable. In block 2, however, the two options in state 2 were rewarded with 80% and 20%, respectively, while both options in state 3 were rewarded with 20%. Therefore, it was more advantageous to choose the option more strongly associated with state 2 in the first-stage. In the following blocks, the advantageous choice in the first stage (“state 1”) alternated as the reward probabilities of the options were switched between two states of the stage 2 after each block transition.

Prior to the experiment, the participants were informed that the reward probabilities for different choices in second stage would change, and that the probabilities of the transitions from the first state to different states in the second stage were fixed throughout the experiment. A practice session was given to familiarize the participants with the structure of the task. The practice session comprised of thirty trials, with five trials in each block.

### Behavioral analyses

A series of two-tailed t-tests were used to examine whether there were differences in task performance between the two conditions. As different measures of performance, we analyzed the average response time to make a choice in the first stage, total points (cumulated reward), and overall probability of selecting the advantageous option (the option more strongly associated with “state 2” in block 2, 4, and 6, and the option more strongly associated with “state 3” in block 3 and 5) in the first stage. Next, a mixed-design ANOVA with outcome type (rewarded or unrewarded), and transition type (common or rare) as within-subjects factors, and treatment (stress or control condition) as between-subjects factors was used to examine whether staying probabilities (the probability of choosing the same option as in the preceding trial) in the first-stage varied significantly with stress, reward on previous trial, and transition type in previous trial. The data were analyzed using the IBM SPSS statistics 21 software.

### Computational modeling

We used a RL model to characterize the trial-by-trial choice dynamics. Various different RL algorithms have been proposed to predict the reward from each option. In this study, we adopted the modified version of the Q-learning model since it performed better than the standard RL model to account for choice behaviors [23]. In the Q-learning model, action values are updated via a simple Rescorla-Wagner (RW) rule [24], and therefore, for a simple binary choice, the value function, V_t_(x), for option x can be updated after each trial t according to the following:

$$v_{t+1}\left(x\right)=V_{t}\left(x\right)+\alpha \left(R_{t}-V_{t}\left(x\right)\right)$$ (1)

where R_t_ means the outcome of the action at the trial t. This is equivalent to the following.

$$V_{t+1}\left(x\right)=\left(1-\alpha \right)V_{t}\left(x\right)+\alpha R_{t}$$ (2)

In the present study, this RL model was modified to quantify model-free and model-based choice behaviors in the first stage of the task [23, 25]. In the model, the action values are updated according to the following:

$$V_{t+1}\left(x\right)=\{\begin{array}{l} \;\left(1-\alpha \right)V_{t}\left(x\right)+\alpha \kappa_{+},\;\mathrm{if}\;\mathrm{it}\;\mathrm{is}\;\mathrm{rewarded},\\ \;\left(1-\alpha \right)V_{t}\left(x\right)+\alpha \kappa_{-},\;\mathrm{if}\;\mathrm{it}\;\mathrm{is}\;\mathrm{unrewarded} \end{array}$$ (3)

where α was the learning rate for the selected option. The parameter κ_+_ represented the strength of reinforcement by the reward outcome, and κ___ represented the strength of punishment by the no-reward outcome. In the present study, this model was expanded to update the value function for the choice in the first stage differently depending on the type of state transition in the same trial. Namely, the perturbation term κ was duplicated to reflect the components expected from model-free (κ^mf^) and mode-based (κ^mb^) RL model. For example, if reward occurred after a common transition, the value function of the option participants chose in the first stage (“state 1”) was updated by κ_+_^mf^ + κ_+_^mb^, since in this case, both model-free and model-based algorithms would attribute the positive outcome to the chosen action. By contrast, if reward occurred after a rare transition, the value function for the option selected in the first stage (“state 1”) was updated by κ_+_^mf^, while the value function for the option unselected was updated by κ_+_^mb^, since in this case, model-free algorithms would attribute this positive outcome to the option chosen and the model-based learning would attribute the positive outcome to the option unchosen. Similarly, if reward did not occur after common transition, the value function of the chosen option was updated by κ___^mf^ + κ___^mb^. If there was no reward after a rare transition, the value function for the chosen option was updated by κ___^mf^_,_ while the value function for the other option was updated by κ___^mb^.

We found that for some subjects, the value of α and κ parameters estimated using the above equations were not stable, since the value of κ could increase in order to compensate a vanishingly small value of the learning rate. Therefore, model parameters were estimated using the following equation, which is mathematically equivalent to (3).

$$V_{t+1}\left(x\right)=\{\begin{array}{l} \;\gamma V_{t}\left(x\right)+\Delta_{+},\;\mathrm{if}\;\mathrm{it}\;\mathrm{is}\;\mathrm{rewarded},\\ \gamma V_{t}\left(x\right)+\Delta_{-},\;\mathrm{if}\;\mathrm{it}\;\mathrm{is}\;\mathrm{unrewarded} \end{array}$$ (4)

where, γ = 1 –α and represents a decay (or discount) factor, a weighting parameter given to the previous value estimate, and Δ = ακ, represents the change in the value function determined by the participant’s choice and its outcome [25]. In other words, Δ_+_^mf^, Δ_+_^mb^, Δ___^mf^, and Δ___^mb^ replaced ακ_+_^mf^, ακ_+_^mb^, ακ___^mf^, and ακ___^mb^, respectively. In this RL model, the tendency to switch away from the unrewarded action corresponds to Δ___ < 0 while the tendency to stay with the same action regardless of no-reward corresponds to Δ___ > 0. More specifically, if Δ___^mf^ and Δ___^mb^ are negative, their magnitudes quantify how strongly model-free and model-based RL predict the tendency to switch to a different option after no reward.

The probability of choosing each option was given by the probability from softmax function related to the difference between the value functions. In other words, denoting the first stage actions by a_1_ and a_2_,

$$P_{t}\left(a_{1}\right)=1/\left(1+\exp\left(-\left(V_{t}\left(a_{1}\right)-V_{t}\left(a_{2}\right)\right)\right)\right)$$ (5)

It should be noted that this model does not require any inverse temperature to determine the randomness in the participant’s choices, since this can be changed by the magnitude of other model parameters (Δ’s). This model is similar to the model used in Daw and his colleagues (2011), except that the value functions for unchosen actions decay gradually.

Parameters of the models were estimated separately for each participant. To maximize the log-likelihood of the data for each subject, we used the Nelder-Mead simplex algorithm [26]. We constrained discount factor to lie between zero and one, and allowed four change parameters to float arbitrarily. Model fitting was iterated 500 times with randomly chosen initial values in order to minimize the risk of finding a local but not global optimal solution.

A series of two-tailed t-tests were used to examine whether there were differences in parameter estimates of the RL models between the two conditions. Also, to test whether stress independently influences the learning rate and the weight of model-free & model-based RL, we performed regression on the model-free or model-based parameter estimate with a decay parameter and a treatment (stress vs. control) for each individual. The data were analyzed using the IBM SPSS statistics 21 software.

## Results

### Effects of stress on decision-making performance

We analyzed the choice behaviors of 52 participants (26 in each condition) during the two-stage reversal learning task following either stress-inducing or control treatment. Task performance of each participant is in S1 Table. As expected, participants in the stress condition rated the hand immersion as significantly more stressful (two-tailed t-test: *t*_50_ = 8.61, *p* < 0.001) than participants in the control condition. Average reaction times for choices in the first stage did not differ significantly for stress and control conditions (two-tailed t-test, *t*_50_ = 1.6, *p* = .116). By contrast, total earnings were significantly lower in the stress condition than in the control condition (*t*_50_ = 2.52, *p* = .015). Also, the probabilities of selecting the advantageous option in the first stage were lower in the stress condition than those in the control condition (*t*_50_ = 2.90, *p* = .006).

**Fig 2:**
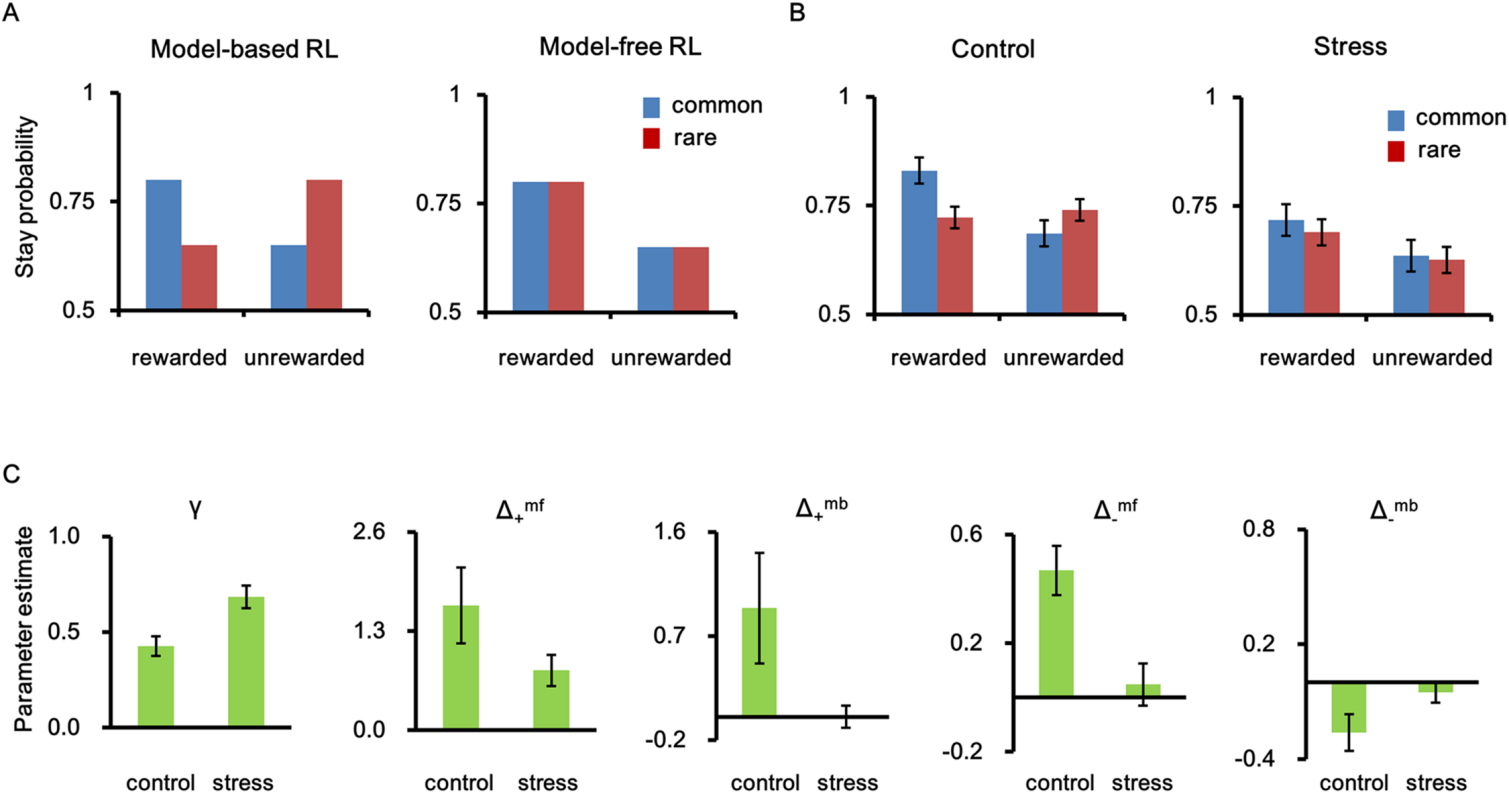
The effects of stress on decision-making. (A) The hypothetic results of stay-shift analysis expected for model-based (left) and model-free (right) reinforcement learning. (B) The behavioral results of stay-shift analysis. Participants’ task performance in control condition showed characteristics of both model-free and model-based influences, while stressed participants showed stronger characteristic of model-free reinforcement learning. The stress × reward × transition interaction, *p* = .004. (C) The results of parameter estimation of a reinforcement learning model. Stress heightened the discount factor, γ (which means stress declined the learning rate) (*p* = .002) and boosted only model-free tendency to switch to a different option after no reward (Δ___^mf^) (*p* < .001). Error bars represent SEM. Δ_+_^mb^ Δ___^mb^, and Δ_+_^mf^ are parameters of the RL model which indicate the model-based tendency after reward, the model-based tendency after no-reward, and the model-free tendency after reward, respectively.

In order to examine how stress might alter the model-free and model-based RL overall, we analyzed the stay-vs.-shift behavior in the first stage. Model-free RL assumes that participants select action solely based on previous choice outcome (reward or no-reward), whereas model-based RL assumes that they choose the optimal actions using their knowledge of the task structure. Therefore, participants relying on model-based RL would tend to stay with the same action even after no reward if this was preceded by a rare transition. By contrast, participants behaving strictly according to model-free RL would choose the same option in the first stage as in the previous trial when the same choice was rewarded in the previous trial, regardless of whether the outcome was preceded by a common or rare transition (Fig 2A). The results from the mixed-design ANOVA revealed a significant main effect of outcome (*F*_(1,50)_ = 18.44, *p* < 0.001), reflecting the pattern predicted for model-free RL. Moreover, a significant reward × transition type interaction (*F*_(1,50)_ = 14.06, *p* < 0.001) showed that there was also a significant effect of model-based RL. More importantly, a significant stress × reward × transition type interaction (*F*_(1,50)_ = 8.86, *p* = 0.004) demonstrated a modulatory role of stress in the coordination of model-free and model-based performance in the task (Fig 2B). Neither stress × reward (*F*_(1,50)_ = 0.06, *p* = 0.81) nor stress × transition type interactions (*F*_(1,50)_ = 0.09, *p* = 0.76) were significant.

### Effects of stress on reinforcement learning model parameters

In order to test multiple factors involved in decision making, and how they are modulated by stress, we applied the RL model that differentiates the model-free and model-based components of action selection. We found that the maximum likelihood estimates of the model parameter for the effect of negative outcome in the model-free learning (Δ___^mf^) was significantly positive in the control condition (*t*_25_ = 5.17, *p* < 0.001) (Table 1). Presumably, the positive value of this parameter indicates that the subjects tended to stay with the same option in the first stage even when the previous outcome was negative. More importantly, the value of this parameter was significantly reduced in the stress condition (*t*_50_ = 3.52, *p* = 0.001) (Table 1, Fig 2C), suggesting that the tendency to avoid the option with the negative outcome was strengthened by stress. There was no significant difference in the model-free effect of positive outcome (Δ_+_^mf^) between the two conditions. Also, stress did not alter the parameters associated with model-based RL (Δ_+_^mb^ and Δ___^mb^). Instead, we found that the discount factor, γ, was significantly higher in the stress compared to the control condition (*t*_50_ = ___3.31, *p* = 0.002). The discount factor determines how rapidly the previous value function is forgotten, and is related to the learning rate α (γ = 1___ α). Thus, it appears that stress boosted only model-free tendency to switch to a different option after no reward and decreased the learning rate, i.e., the ability to incorporate new information into decision-making.

**Table 1:** Best-fitting parameter estimates, shown as median plus quartiles across conditions.

	γ^*^	Δ_+_^mf^	Δ_+_^mb^	Δ___^mf^^**^	Δ___^mb^
**CONTROL**					
**25**^**th**^	0.23	0.70	0.10	0.12	-0.41
**Median**	0.47	1.09	0.33	0.48	-0.14
**75**^**th**^	0.60	1.67	0.80	0.78	0.04
***T***	8.41**	3.30*	2.00	5.17**	-2.74
**STRESS**					
**25**^**th**^	0.56	0.04	-0.23	-0.10	-0.13
**Median**	0.73	0.50	-0.04	0.02	-0.01
**75**^**th**^	0.95	1.06	0.18	0.14	0.11
***T***	11.50**	3.84*	.02	.62	-.89

Notes: Δ_+_^mb^ and Δ___^mb^ are parameters which represent the model-based tendency after reward and no-reward, respectively. Δ_+_^mf^ and Δ_+_^mf^ are parameters which indicate the model-free tendency after reward and no-reward, respectively.

* *p* < 0.01

^**^ *p* < 0.001. T is the *t* value from the paired t-test which was performed to investigate whether each parameter was significantly different from zero.

We performed additional analyses in order to clarify further how the changes in model-free RL were related to stress treatments. First, to test whether stress independently influences the learning rate and the weight of model-free RL after negative outcome, we performed regression on the model-free parameter estimate (Δ___^mf^) with a decay parameter and a treatment (stressed or not) for each subject. The results confirmed that the effect of stress on model-free RL after negative outcome (Δ___^mf^) was significant (B = -0.433, SE = 0.133, *β* = -0.459, *p* = 0.002) even after controlling for the effect of stress on the learning rate (B = 0.049, SE = 0.218, *β* = 0.032, *p* = 0.822). Second, we conducted the ANCOVA with the probability of the advantageous action as a covariate. In the present study, a reversal learning component was incorporated into the two-stage decision-making task [22]. During a two-stage decision task with reversal, subjects with model-free tendency, who simply choose the advantageous option in each block, might appear to have a model-based tendency [27]. The ANCOVA results showed that the effect of stress on the strength of model-free RL after receiving negative outcome was significant even after controlling for the probability of the advantageous action (*F*_*(1*,*49)*_ = 8.01, *p* = 0.007). Taken together, these results suggest that stress increased the contribution of model-free RL only after negative outcomes.

## Discussion

In this study, we found that stress impaired the reward-seeking behavior and demonstrated that the inferior performance under stress might be due to at least two different mechanisms. First, stress increased the influence of the model-free reinforcement learning, particularly the likelihood of switching to an alternative choice when the previous choice led to an undesirable outcome. Second, stress decreased the learning rate, namely, the degree to which new information is incorporated into trial-by-trial decision making. These findings suggest that maladaptive choice behavior under stress might be attributable to both a slower learning rate and the strengthening of model-free RL after a negative outcome.

It has not been investigated clearly whether stress leads to more habitual behaviors by selectively weakening the process of goal-directed behaviors, by merely strengthening the process of habit, or both. In order to investigate the effect of acute stress on the distinct contributions of habit and goal-directed processing, recent researches have tried to use computational modeling for reinforcement learning (RL) to separate the habit and goal-directed processing into two RL algorithms, model-free and model-based, respectively. In previous computational studies, however, the effects of acute stress on the two RL were inconsistent [28, 29]. Otto and his colleagues showed that stress-related physiological (cortisol) response was negatively correlated with model-based but not model-free contributions. However, their study did not demonstrate the effect of stress on decision making itself. Also, Radenbach and his colleagues reported the effect of stress on the ratio of model-based RL to model-free RL, but without clearly separating out the effects of stress on model-based RL from those of model-free RL[29]. Thus, how acute stress facilitates habit or model-free choice behavior remained incompletely understood.

In this study, we investigated the effects of stress on model-free and model-based RL using a 2-step decision task incorporating the reversal learning paradigm and showed that stress increased the model-free RL without altering the strength of model-based RL. These results suggested that stress-enhancement of habit behavior may not be merely compensatory byproduct of impaired model-based RL behavior. Also, habitual processing might be strengthened by stress because stress disrupts inhibition of the model-free processing which could be a default model of RL[30]. Furthermore, we differentiated the model-free tendency to make a shift following no-reward (lose-switch) and to stay following a reward (win-stay), and showed that stress increased the model-free RL after no-reward selectively without affecting the model-free RL after reward. These results suggest that stress may disproportionately boost the neural processing of decision-making involved in model-free learning from negative outcomes. Our findings are consistent with previous studies showing there are separate neural processing for reinforcement and punishment [16, 17, 31].

Although the 2-step decision task has been designed to distinguish model-free and model-based RL, a recent study revealed that the original task does not lead to significant difference in performance (points or income) predicted by model-based vs. model-free RL approach, through a computational simulation [27]. Therefore, we incorporated a reversal learning paradigm into the original task, which produced more consistent difference in the performance for the two RL strategies. However, in a reversal learning task, decision-makers can learn that there are two distinct latent states of the task and rely on such inference about the current latent state to make their choices [32, 33]. The decision-maker who infers and uses latent state of the task could outperform a standard model-free RL and looks like a model-based decision-maker, even without using the knowledge of the transition structure linking the first actions to the states of the second stage [34]. In this study, stress enhanced only model-free RL without impairing model-based RL. We conducted the ANCOVA with the probability of selecting the advantageous action as a covariate, which would reflect the tendency to make choices based on the inference about a latent state. The results from this analysis showed that the effect of stress on the strength of model-free RL after receiving negative outcome was significant even after controlling for the probability of the advantageous action. Therefore, stress might increase the contribution of model-free RL regardless of its effect on the ability to make choices based on the inferred state of the environment.

Also, we found that stress decreased the learning rate during a reward-based choice task. In the RL model, the learning rate reflects how quickly the valuation of selected action is updated by the difference between the prediction and the actual outcome, referred to as the prediction error [2]. Therefore, it represents how rapidly new information from the environment is incorporated in subsequent actions [35–37]. For adaptive decision making, it is critical to utilize new information efficiently and to avoid maladaptive perseverative behaviors when faced with environmental change. Decision-makers with low learning rate would fail to switch their behaviors flexibly in response to unexpected changes in the real world. It is possible that a decrease in learning rate under stress may be an important factor contributing to stress-induced alteration in RL. However, we could not examine the effect of stress on the distinct learning rate of model-free and model-based RL, because we estimated a single learning rate from observed choices and rewards for each subject. Further investigations are necessary to clarify whether stress changes learning rate during both model-free and model-based RL.

## Conclusions

This study characterized the effect of stress on adaptive decision making, by providing participants with a changing environment where their choice behaviors were modeled in a computational framework of reinforcement learning. We found that stress facilitated the habitual, model-free RL process to shift away from unrewarded action, and that it also interrupted the subjects from incorporating new information into their subsequent choices. These findings provide insight as to the mechanism by which stress diminishes the ability to behave flexibly in reward-based decision making, and have significant implications for understanding and treating stress-related maladaptive conditions characterized by enhanced habit behavior such as addiction and impulse control disorders.

## Supporting information

S1 Table Task performance for each subject. (XLSX)
